# The E-Subgroup Pentatricopeptide Repeat Protein Family in *Arabidopsis thaliana* and Confirmation of the Responsiveness *PPR96* to Abiotic Stresses

**DOI:** 10.3389/fpls.2016.01825

**Published:** 2016-12-05

**Authors:** Jia-Ming Liu, Juan-Ying Zhao, Pan-Pan Lu, Ming Chen, Chang-Hong Guo, Zhao-Shi Xu, You-Zhi Ma

**Affiliations:** ^1^Key Laboratory of Molecular Cytogenetics and Genetic Breeding of Heilongjiang Province, College of Life Science and Technology, Harbin Normal UniversityHarbin, China; ^2^Key Laboratory of Biology and Genetic Improvement of Triticeae Crops, Ministry of Agriculture, Institute of Crop Science, Chinese Academy of Agricultural Sciences/National Key Facility for Crop Gene Resources and Genetic ImprovementBeijing, China

**Keywords:** *Arabidopsis thaliana*, pentatricopeptide repeat (PPR) proteins, microarray analysis, seed imbibition, seed development stage(s), flower development processes, mitochondria, abiotic stresses

## Abstract

Pentatricopeptide repeat (PPR) proteins are extensive in all eukaryotes. Their functions remain as yet largely unknown. Mining potential stress responsive PPRs, and checking whether known PPR editing factors are affected in the stress treatments. It is beneficial to elucidate the regulation mechanism of PPRs involved in biotic and abiotic stress. Here, we explored the characteristics and origin of the 105 E subgroup PPRs in *Arabidopsis thaliana*. Phylogenetic analysis categorized the E subgroup PPRs into five discrete groups (Cluster I to V), and they may have a common origin in both *A. thaliana* and rice. An *in silico* expression analysis of the 105 E subgroup PPRs in *A. thaliana* was performed using available microarray data. Thirty-four PPRs were differentially expressed during *A. thaliana* seed imbibition, seed development stage(s), and flowers development processes. To explore potential stress responsive PPRs, differential expression of 92 PPRs was observed in *A. thaliana* seedlings subjected to different abiotic stresses. qPCR data of E subgroup PPRs under stress conditions revealed that the expression of 5 PPRs was responsive to abiotic stresses. In addition, *PPR96* is involved in plant responses to salt, abscisic acid (ABA), and oxidative stress. The T-DNA insertion mutation inactivating *PPR96* expression results in plant insensitivity to salt, ABA, and oxidative stress. The PPR96 protein is localized in the mitochondria, and altered transcription levels of several stress-responsive genes under abiotic stress treatments. Our results suggest that *PPR96* may important function in a role connecting the regulation of oxidative respiration and environmental responses in *A. thaliana*.

## Introduction

Pentatricopeptide repeat (PPR) proteins are encoded by a large gene family in terrestrial plants. This family has 450 members in *Arabidopsis thaliana*, 477 members in rice, and 486 members in foxtail millet (*Setaria italica*) (Schmitz-Linneweber and Small, 2008; Liu et al., 2016). Non-plant organisms contain very few PPRs (Lurin et al., 2004; Andrés et al., 2007). For example, PPRs are virtually absent from prokaryotes (Pusnik et al., 2007), and the yeast, *Drosophila* and human genomes are predicted to contain only 5, 2, and 6 PPR genes, respectively, and it is clear that the family has expanded greatly in terrestrial plants (O'Toole et al., 2008; Liu et al., 2016). The PPR gene family was identified serendipitously over a decade ago as a result of bioinformatic analyses of the incomplete *A. thaliana* genome sequence (Small and Peeters, 2000). The higher plant PPR proteins are characterized by tandem arrays of a degenerate 35-amino acid repeat motif and have functions in RNA or DNA modification, acting through sequence-specific binding (Saha et al., 2007). In *A. thaliana*, this family can be split into two subfamilies based on the structure of the repeated motif: the P subfamily and the PLS subfamily. Members of the P subfamily contain the canonical P motif common to all eukaryotes, while members of the PLS subfamily contain the P motif as well as two P motif-derived variants, the short (S), and the long (L) motifs. Based on the presence of conserved domains in the C-terminal region, the PLS subfamily can be further divided into the PLS, E (E/E+), and DYW subgroups (Lurin et al., 2004; Andrés et al., 2007). The majority of plant PPR proteins are predicted to be localized to mitochondria or chloroplasts. PPR proteins characterized to date have functions in a wide range of physiological and developmental processes, including cytoplasmic male sterility, photosynthesis, respiration, and embryogenesis. A few of these proteins have been shown to play roles in post-transcriptional processes associated with RNA in plant organelles (Zsigmond et al., 2008; Liu et al., 2010; Sung et al., 2010; Laluk et al., 2011; Murayama et al., 2012; Ye et al., 2012).

There have been very few functional studies of PPR proteins relating to biotic and abiotic stress-response mechanisms in higher plants. To date, of the 450 predicted PPR proteins, only GUN1, PPR40, ABO5, PGN, AHG11, SLG1, and MEF11/LOI1 have been implicated with *A. thaliana* defense biotic or abiotic stress response (Koussevitzky et al., 2007; Zsigmond et al., 2008; Liu et al., 2010; Laluk et al., 2011; Murayama et al., 2012; Yuan and Liu, 2012). GUN1 is implicated with plastid-to-nucleus retrograde signaling, regulation of nuclear gene *ABI4* expression, and photooxidative stress responses (Koussevitzky et al., 2007). *A. thaliana* PPR40 is implicated with mitochondrial oxidative respiration that also contributes to abiotic stress tolerance (Zsigmond et al., 2008). ROS accumulation was increased and some stress-responsive gene expression were altered in the *ppr40* mutant grew in the culture medium contained ABA and salt (Zsigmond et al., 2008). ABO5 was required for *cis*-splicing of the mitochondrial *nad2* intron 3, and altered the expression of several stress-responsive and nuclear-encoded genes to affect the ABA signaling pathway (Liu et al., 2010). The PGN functions in the regulation of reactive oxygen species (ROS) homeostasis by regulating RNA editing events in mitochondria during abiotic and biotic stress responses may occur through the regulation of mitochondria-nucleus retrograde signaling (Laluk et al., 2011). AHG11 regulates the *nad4* transcriptional level and thus led to changes in oxidative levels by controlling RNA editing events in mitochondria and affecting plant responses to ABA (Murayama et al., 2012). SLG1 regulates the *nad3* transcript by regulating RNA editing events in mitochondria and affecting the expression of genes involved in the alternative respiratory pathway (Yuan and Liu, 2012). MEF11/LOI1 is involved in mitochondrial RNA editing, and regulates biosynthesis of isoprenoids, metabolites known to affect defense gene expression in response to wounding and pathogen infection (Kobayashi et al., 2007; Tang et al., 2010). The prevalence of chloroplast- and mitochondrial-physiology in the findings of functional studies of many PPR proteins suggests that many of these proteins may play roles in regulating oxidation balance in cellular redox under different types of stress (Lurin et al., 2004; Andrés et al., 2007). Of the seven PPR proteins, GUN1, PPR40, and ABO5 belong to P subfamily, PGN, AHG11, and SLG1 belong to E subgroup, and MEF11/LOI1 belong to DYW subgroup which also contains E/E+ motif. Four members contain an E/E+ motif (MEF11/LOI1, PGN, AHG1, and SLG1), and those PPR proteins contain E/E+ motif (E and DYW subgroups) relating to biotic and abiotic stress-response by regulating RNA editing events in mitochondria and chloroplast (Kobayashi et al., 2007; Tang et al., 2010; Laluk et al., 2011; Murayama et al., 2012; Yuan and Liu, 2012). It is thought that the E/E+ motif might have a common function among trans-factors of RNA editing in chloroplasts or mitochondria (Okuda et al., 2007). RNA editing is important for plants to maintain functional chloroplast and mitochondrial transcripts. As a vital component, PPR protein provides specificity of each editing event. However, very little has been done to study how editing is regulated under different stresses and whether it can be used as a coping mechanism. It would be interesting to the field to check whether known PPR editing factors are affected in the stress treatments. However, there have been very few functional studies of PPR proteins relating to biotic and abiotic stress response mechanisms in higher plants.

To explore potential stress responsive PPR proteins, and checking whether known PPR editing factors are affected in the stress treatments. Here, we explored the characteristics of 105 E subgroup PPR proteins in *A. thaliana*, including chromosomal location and phylogenetic relationships. A comprehensive *in silico* expression analysis of the E subgroup PPR genes at several development stages of *A. thaliana*, including seed imbibition, seed development, and flowers has been performed using available microarray data. The expression patterns under stress conditions showed that five E subgroup PPR genes are responsive to abiotic stresses, and finally confirmed that PPR96 is involved in abiotic stresses. In addition, we studied the function of *A. thaliana* PPR96 in finer detail. A fusion of the protein encoded by this gene with GFP was localized to the mitochondria. The expression of *PPR96* was found to be responsive to salt stress, oxidative stress, and ABA treatments.

## Materials and methods

### Database searches for E subgroup PPR genes in *A. thaliana*

The gene and protein sequences of the *A. thaliana* E subgroup PPRs were acquired from TAIR (http://www.arabidopsis.org/); those of rice E subgroup PPRs were acquired from TIGR (http://rice.plantbiology.msu.edu/). The gene microarray data for *A. thaliana* were obtained from the NASCArrays server (http://bar.utoronto.ca/). Data for the following *A. thaliana* tissue/organs and developmental stages were analyzed: seed imbibition, roots, shoots and stems, leaves, flowers, and seed (Winter et al., 2007). Differential expression was defined according to the previous description: a gene as differentially expressed at a given stage only if the expression level of the gene at that stage was significantly higher (more than 2-fold) than the levels at all the other stages (Jain et al., 2007).

### Identification of E subgroup PPR genes and evolutionary analyses

To explore the evolutionary relationship of E subgroup PPR genes in the flowering plants, we identify probable candidate eudicot model plant *A. thaliana* and monocots model plant rice E subgroup PPRs. The PPR domain (Pfam: PF01535) and the E/E+ motif (Lurin et al., 2004) were submitted as queries in BLASTP (*E*-value ≤ 10) searches of the *A. thaliana* and rice genome databases. A total of 105 *A. thaliana* and 138 rice putative E subgroup PPRs were obtained after manually filtering out repeated sequences. The amino acid sequences of the putative E subgroup PPRs were imported into MEGA5 (Tamura et al., 2011), and multiple sequence alignments were performed using ClustalW with a gap open penalty of 10 and a gap extension penalty of 0.1 (Thompson et al., 1997). The alignment was then used to produce an unrooted phylogenetic tree based on the neighbor-joining method (Saitou and Nei, 1987), and Poisson model was used as the amino acid substitution model; after bootstrap analysis with 500 replications, the final tree was generated.

### Exon-intron structure and phylogenetic analysis

Manual curation and assessment of the numbers of introns and the exon-intron positions of the genes was based on comparing the full-length cDNA of the putative E subgroup PPR genes with their corresponding genomic sequences in *A. thaliana*. Physical mapping of the genes encoding the putative E subgroup PPRs onto the *A. thaliana* genome was performed by conducting BLASTP searches of the sequences against the Phytozome database adopting the default settings. The map was displayed using MapInspect software.

### Reverse transcription-PCR (RT-PCR) and quantitative real-time PCR (qPCR)

Total RNA from leaves of *A. thaliana* (Col-0 and *ppr96* mutants) was extracted using TRIzol reagent (Invitrogen) according to the manufacturer's instructions, and treated with RNase free DNase I (2 U μl^−1^, Invitrogen). The quality and purity of the RNA preparations were determined by measuring the OD260/OD280 absorption ratio (1.8–1.9), and the integrity of the preparations was determined according to the previous studies (Lata et al., 2014). DNase-treated RNA was examined by PCR to ensure complete DNA removal. Subsequently, cDNA synthesis, and RT-PCR were conducted as previously described (Xu et al., 2007), *A. thaliana Act2* gene was used as an internal control. qPCR analysis for the examination of the expression of the putative E subgroup PPR genes was carried out according to the previous description (Liu et al., 2016), PCR was performed based on three technical replicates for each of the biological duplicates. *Act2* was used as an internal control to normalize the expression data. qPCR data analysis was done according to previous studies (Livak and Schmittgen, 2001; Lata et al., 2014). All primers used in the study are listed in Table S1.

### Plant materials and multiple stress treatments

Seeds of wild type *A. thaliana* (Columbia, Col-0) and mutant lines *ppr96-1* (SALK_045553C) and *ppr96-2* (SALK_121064) were surface sterilized with bleach and thoroughly washed three times with sterile water before incubation in a growth chamber following 3 days of cold treatment. For the stress treatments, 4-week-old plants were exposed to solutions containing, variously, 200 mM NaCl, 50 μM ABA, or 10 mM H_2_O_2_. Unstressed plants were maintained as controls. All plant materials were harvested and stored at −80°C.

### Examination of T-DNA insertion mutants

The T-DNA insertion site in the *ppr96* mutants was confirmed by PCR with the gene-specific primers in Table S1.

### Seed germination and root growth assays

For the germination assays, Col-0 and *ppr96* seeds were placed on ½ MS medium containing different contents of exogenous ABA (0.5–1 μM), or NaCl (0–200 mM). For the root growth assays, 7-day-old seedlings were grown on vertical agar plates in the presence or absence of ABA (5 or 10 μM) or NaCl (80 or 120 mM). Root lengths were measured after 14 days.

### Stomatal opening assay and measurement of chlorophyll content

Stomatal opening assays were performed with epidermal peels from rosette leaves of 4- to 6-week-old plants according to the previous description (Leymarie et al., 1998). Stomatal pores were measured according to previously-described methods (Li et al., 2012). For the measurement of chlorophyll content, samples (0.05–0.1 g) of 3-week-old *in vitro* grown control and treated seedlings were homogenized in liquid nitrogen and extracted with 80% (v/v) acetone for 2 h. The homogenate was centrifuged at 15,000 × g for 10 min. Absorption of the extracts was measured at 663 and 645 nm and the chlorophyll A and chlorophyll B concentrations were calculated according to Lichtenthaler (1987).

### Subcellular localization

The coding region of *PPR96* was fused to the N-terminal end of GFP under control of the CaMV 35S promoter. For transient expression assays, ~4 × 10^4^ *A. thaliana* mesophyll protoplasts were isolated and transfected with 15 μg of *35S*::*PPR96* plasmid. The empty GFP vector was used as a control. Transfected protoplasts were incubated in darkness at 25°C for 16 h (Xu et al., 2007; Liu et al., 2013). Transfected *A. thaliana* protoplasts were stained with a mitochondria-specific dye [MitoTracker Orange (Invitrogen catalog no. M7510)] (Jiang et al., 2006) and then observed with 488 and 543 nm illumination using a Zeiss LSM700 microscope.

## Results

### Identification of E subgroup PPR genes and phylogenetic analysis

In the *A. thaliana* genome, 450 putative PPR genes were identified, including 106 E subgroup PPR genes. (Lurin et al., 2004). However, *At1g06145* was listed as obsolete in TAIR (http://www.arabidopsis.org/). Finally, a total of 105 E subgroup PPR genes in the genome of *A. thaliana* were identified using techniques described in the Materials and Methods section of this paper. The PHYTOZOME locus, subclass, open reading frame ORF length, predicted protein length, intron number, chromosomal location, and predicted protein targeting of each of these 105 E subgroup PPR genes is listed in Table S2. The predicted polypeptide lengths of E subgroup PPR proteins varied widely, ranging from 429 to 1028. EXPASY analysis suggested that the predicted E subgroup PPR protein sequences have great variations in both isoelectric point (p*I*) values (ranging from 5.23 to 9.11) and molecular weight (ranging from 46.921 to 111.541 kDa; Table S1).

**Figure 1:**
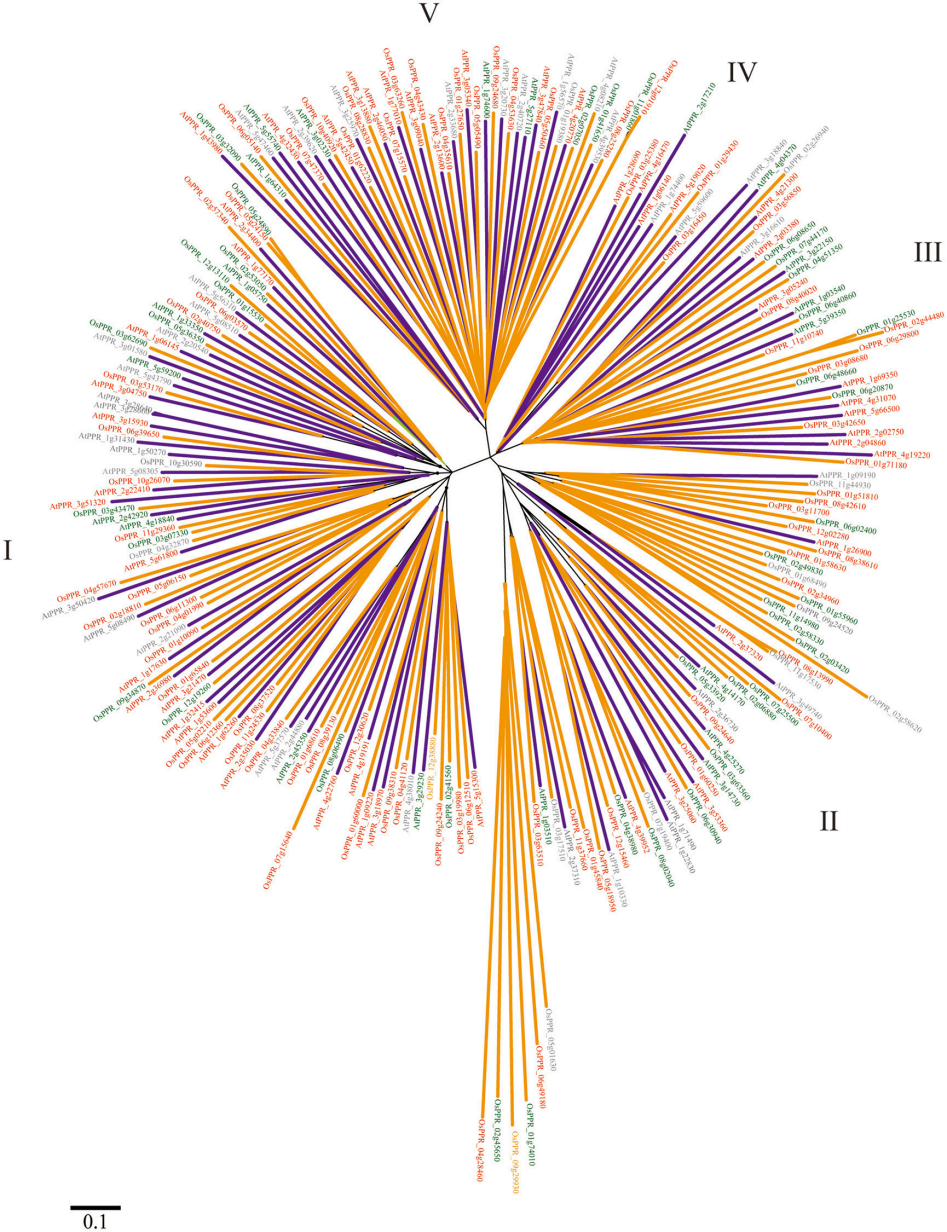
**NJ distance tree of E subgroup PPR proteins ***A. thaliana*** and rice**. The tree is presented radially so that distances from the center represent cumulative branch lengths. Terminal branches and labels are colored to indicate: species (*A. thaliana*, blue; rice, orange) and predicted organelle targeting (mitochondria, red; plastids, green; and cytoplasm, gray).

To evaluate the phylogenetic relationships among the E subgroup PPR genes in *A. thaliana*, a phylogenetic analysis of 105 *A. thaliana* and 138 rice predicted E subgroup PPR sequences was performed by generating a neighbor-joining phylogenetic tree (Figure 1). The phylogenetic analysis categorized the E subgroup PPR proteins into five discrete groups (Cluster I to V), containing, respectively, 45, 16, 14, 7, and 23 predicted proteins (Figure 1). Many of the internal branches had high bootstrap values, indicating statistically reliable pairs of possible homologous proteins.

### Gene structure and chromosomal distribution

The intron numbers present within the ORF of each of the E subgroup PPR genes in *A. thaliana* were determined by analysis of their exon-intron organization. The large majority of the E subgroup PPR genes contain very few introns. Ninety-two E subgroup PPR genes contain no introns, 11 contain 1 intron, 3 and 4 introns were found in *At3g50420* and *At2g42920*, respectively. It has been proposed that the gene families which are lack of intron can evolve rapidly, and the ways are either by gene duplication or reverse transcription/integration (Lecharny et al., 2003; Lurin et al., 2004; Jain et al., 2006).

**Figure 2:**
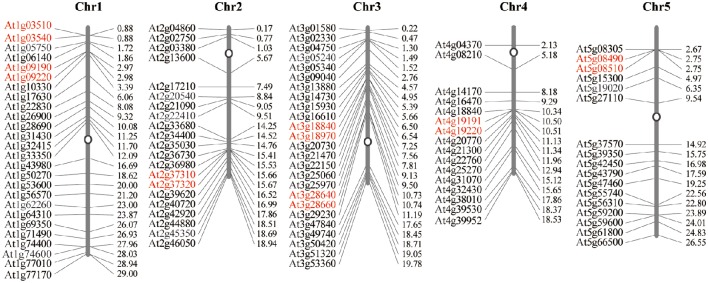
**Distribution of the 105 E subgroup PPR genes onto the five ***A. thaliana*** chromosomes**. Graphical (scaled) representation of physical locations for each E subgroup PPR gene on the five *A. thaliana* chromosomes (numbered 1–5). Tandem duplicated genes on a particular chromosome are depicted by scarlet letters. Chromosomal distances are given in Mb.

The 105 E subgroup PPR genes are unevenly distributed across all 5 of the *A. thaliana* chromosomes. Chromosome 1 of *A. thaliana* contains the highest number of the E subgroup PPR genes [26(24.76%)], while the lowest number are on chromosome 4 [16(15.23%)]. The exact position (in bp) of each E subgroup PPR gene on the *A. thaliana* chromosomes is available at PHYTOZOME and is represented diagrammatically in Figure 2 (the exact position in bp is given in Table S2).

### *In silico* microarray analysis of the E subgroup PPR genes during seed imbibition, seed, and flower development

To get gene expression profiling of E subgroup PPR genes in *A. thaliana*, microarray analysis was performed using available microarray data, which were downloaded from the NASCArrays server (http://bar.utoronto.ca/). The log signal values of *A. thaliana* corresponding tissues/organs and developmental stages of the 105 E subgroup PPR genes represented on the array were extracted.

**Figure 3:**
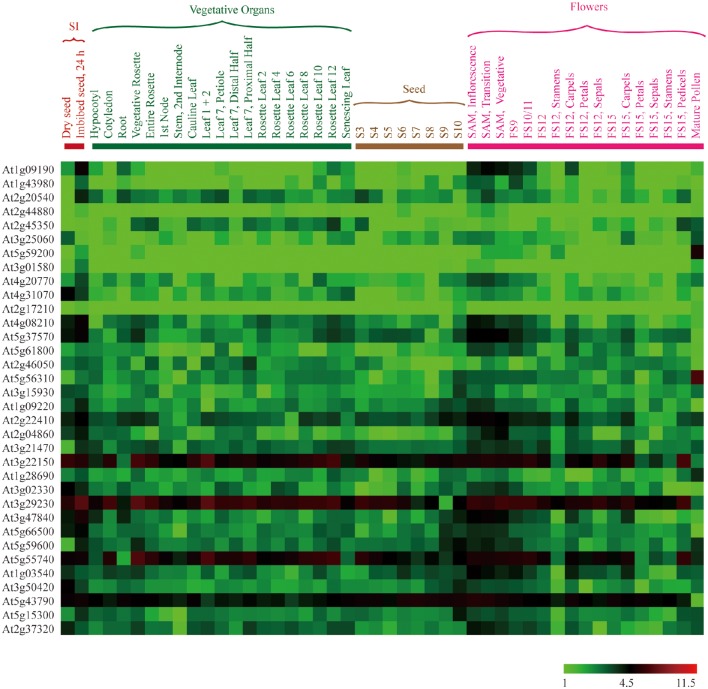
**Expression profiles of ***A. thaliana*** E subgroup PPR genes differentially expressed during seed imbibition, seed development stage(s), and flowers**. The average log signal values of E subgroup PPR genes in various tissues/organs and developmental stages (mentioned at the top of each lane) are presented by cluster display. The color scale (representing log signal values) is shown at the bottom.

The average log signal values for all of the 105 E subgroup PPR genes are given in Table S3, and visualized in a hierarchical cluster in Figure S1. The results show that the majority of E subgroup PPR genes are expressed in at least one of the *A. thaliana* vegetative organs and/or stages of development that we downloaded the data for. Subsequently, differential expression analysis was performed to identify the E subgroup PPR genes with the highest expression among seed imbibition, the seed development stage(s), and flowers (Figure 3). Differential expression was defined according to the description of Jain et al. (2007). This analysis revealed that a total of 25, 23, and 34 E subgroup PPR genes were differentially expressed in at least one of the stages of seed imbibition, the seed development stage(s), and flowers, respectively (Figure 3, Table S4).

### Microarray analysis of E subgroup PPR genes under abiotic stresses conditions

**Figure 4:**
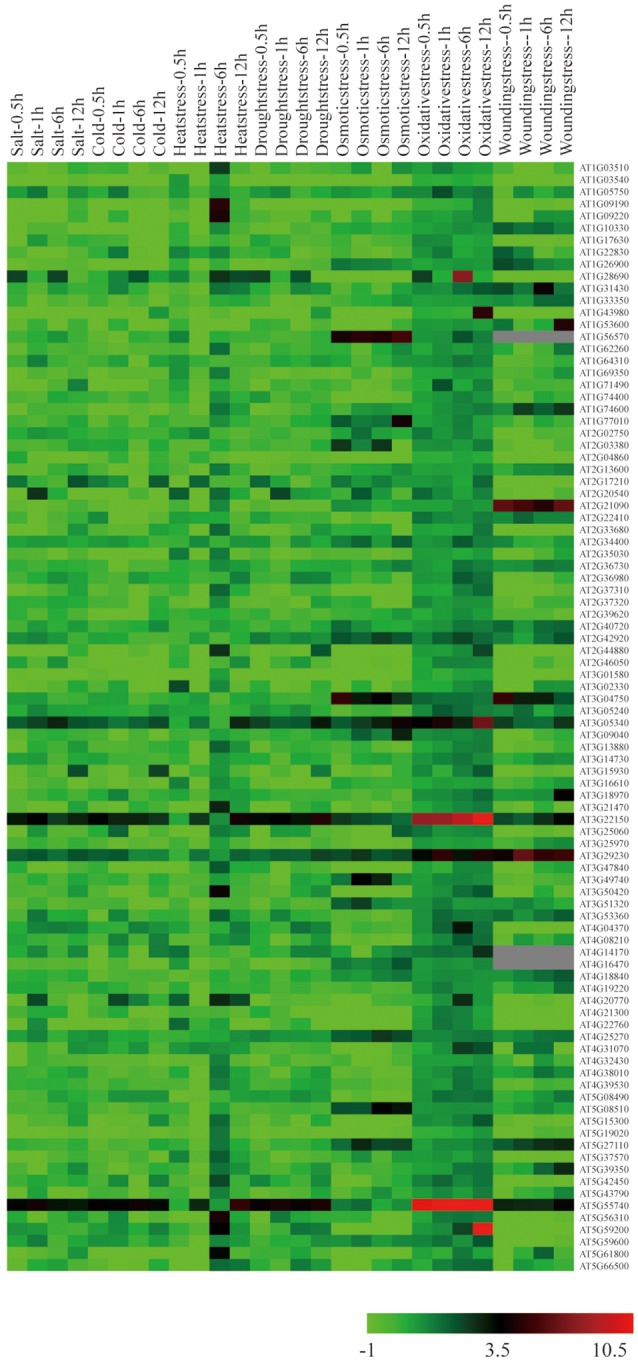
**Expression profiles of E subgroup PPR genes differentially expressed under abiotic stress conditions**. The values of E subgroup PPR genes under control (untreated) and various stress conditions (mentioned at the top of each lane) were presented by cluster display (values are given in Table S5). The color scale (representing signal values) is shown at the bottom.

To examine expression patterns of *A. thaliana* E subgroup PPR genes under abiotic stress treatments, and mine potential stress responsive E subgroup PPR genes, microarray analysis was performed using available NASCArrays data (http://bar.utoronto.ca/) for RNA from *A. thaliana* seedlings subjected to salinity, cold, heat, drought, osmotic, oxidative, and wounding treatments for 0.5, 1, 6, and 12 h. We were able to identify 92 E subgroup PPR genes that were differentially expressed under one or more of these stress conditions (Figure 4 and Table S5). 84, 74, 88, 83, 62, 57, and 72 genes were differentially expressed under the salt, cold, heat, drought, osmotics, oxidative, and wounding treatments, respectively (Figure 4), and the differentially expressed genes were labeled by different background colors in Table S5. Twenty-seven E subgroup PPR genes were differentially expressed under all of the abiotic stress treatments (Table S5). Microarray analysis results showed that a large number of the E subgroup PPR genes were differentially expressed under the abiotic stress treatments, which may be due to the E/E+ motif of E subgroup PPR proteins which are implicated with the site-specific RNA editing events in plant mitochondria or chloroplasts (Okuda et al., 2007) to alter oxidation balance *in vivo* when plants grow under the abiotic and biotic stress conditions (Zsigmond et al., 2008; Liu et al., 2010; Laluk et al., 2011; Murayama et al., 2012; Yuan and Liu, 2012).

### Verification of microarray data using qPCR

**Figure 5:**
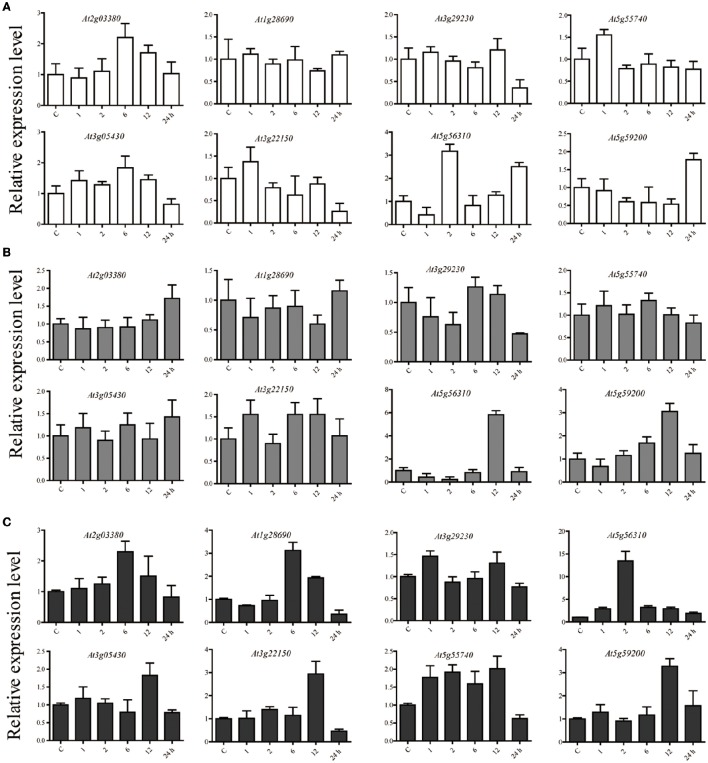
**The relative expression levels of 8 candidate E subgroup PPR genes analyzed using qPCR under (A)** salinity stress, **(B)** ABA treatment, and **(C)** oxidative stress for 1, 2, 6, 12, and 24. The relative expression level of each gene was calculated relative to their expression in untreated control samples. *Act2* was used as an internal control to normalize the expression data. The error bars represent the standard deviation calculated based on three technical replicates for each of the biological duplicates.

We used qPCR analysis to verify that the microarray results of interest. E subgroup PPR proteins were involved in biotic and abiotic stress-response by regulating RNA editing events in mitochondria and chloroplast, and many of these proteins may play roles in regulating oxidation balance *in vivo* elicited by different types of stress (Lurin et al., 2004; Andrés et al., 2007; Kobayashi et al., 2007; Tang et al., 2010; Laluk et al., 2011; Murayama et al., 2012; Yuan and Liu, 2012). Considering this, 8 *A. thaliana* E subgroup PPR genes based on the expression level under oxidative stress in the microarray results were selected for further verification. *A. thaliana* plants were subjected to salinity, ABA, and oxidative stress for 1, 2, 6, 12, and 24 h, the unstressed plants were maintained as controls in each time point, and gene expression was analyzed with qPCR. In summary, the qPCR analysis showed that all of the candidate E subgroup PPR genes had variations in their expression patterns in response to one or more stresses relative to their expression in untreated control samples (Figure 5). The expression of 2 genes were up-regulated (>2-fold) by salt stress (*At2g03380*, and *At5g59200*), 2 were up-regulated by ABA (*At5g56310*, and *At5g59200*), and 5 were up-regulated by oxidative stress (*At2g03380, At1g28690, At5g56310, At3g22150*, and *At5g59200*). The expression of *At2g03380* was up-regulated under both salt and oxidative stress treatments. *At5g59200* expression was also up-regulated under both ABA and oxidative stress conditions. Notably, the expression of *At5g56310* was up-regulated under salt, ABA, and oxidative stress conditions. We found that the expression of three *A. thaliana* E subgroup PPR genes (*At3g05340, At3g29230*, and *At5g55740*) was unchanged by either stress. The results of qPCR broadly consistent with the microarray analysis, and showed that *At2g03380* (*PPR96*), *At5g59200* (*OTP80*), and *At5g56310* (*MEF21*) are responsive to abiotic stresses and ABA.

### The expression of the *PPR96* gene in *A. thaliana* and *ppr96* mutants insensitivity to salt stress

**Figure 6:**
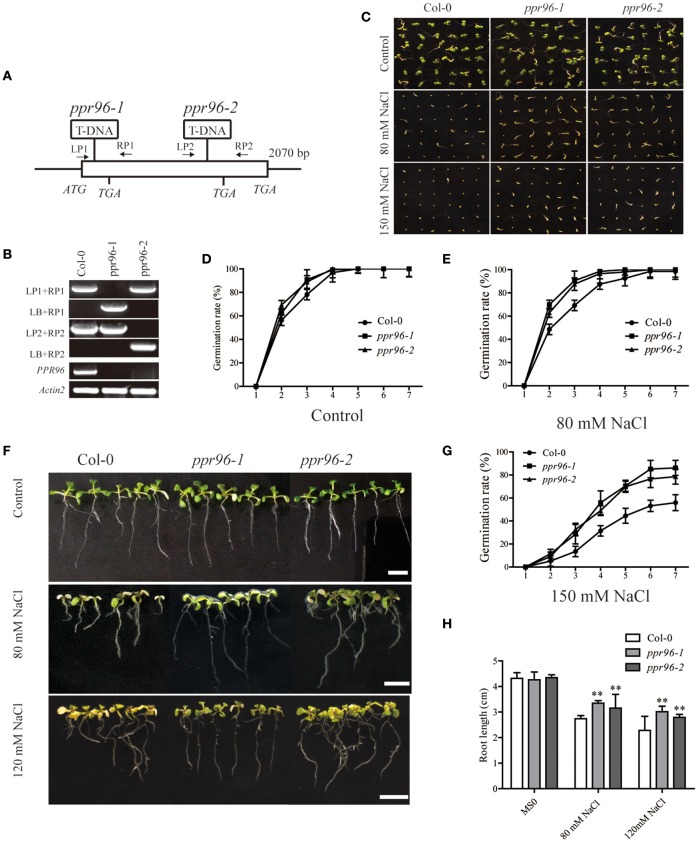
**Response of the ***ppr96*** mutants to salt stress. (A)** and **(B)** Verification of the T-DNA insertion in the *ppr96* mutants. Positions of T-DNA insertions in the *At2g03380* gene, and *PPR96* expression was not detected in mutants plants. **(C–F)** Germination rates of seeds after 7 days in the presence or absence of NaCl. **(G)** Phenotypic comparison of root lengths of plants grown on MS medium with or without NaCl. Images were recorded on day 7 after the transfer of 7-day-old seedlings from ½ MS medium to plates containing NaCl. Bars = 1 cm. **(H)** Effect of different NaCl concentrations on root growth in Col-0 and mutant plants. Data represent means ± SD (*n* = 30). Student's *t*-tests were used to generate the *P*-values. ^*^P < 0.05; ^**^P < 0.01.

According to the results of our qPCR analysis, *At2g03380* (*PPR96*), *At5g59200* (*OTP80*), and *At5g56310* (*MEF21*) were selected for further study whether they respond to abiotic stresses. We obtained mutants for these genes from the *Arabidopsis* Biological Resource Center (ABRC, http://abrc.osu.edu) and verified that the *ppr96* mutants seedlings were insensitive to salt, abscisic acid, and oxidative stress as compared to Col-0 plants. Mutants of the other two genes had no obvious phenotype to abiotic stresses treatment (data not shown). We confirmed that the *ppr96* mutants by a tandem PCR, including *ppr96-1* (SALK_045553) and *ppr96-2* (SALK_121064). The *ppr96-1* mutant has a T-DNA insertion caused a target site addition of 1 bp in the 66 bp downstream of the *ATG* codon, resulting in the presence of a stop codon in the 96 bp downstream of the predicted *ATG* codon (Figure 6A). The *ppr96-2* mutant has a T-DNA insertion caused a target site deletion of 1 bp and was localized 1455 bp downstream of the predicted *ATG* codon (Figure 6A). The T-DNA insertion in *ppr96* disrupts the expression of *PPR96*; RT-PCR analysis revealed that no *PPR96* transcripts were detectable in *ppr96* mutants (Figure 6B).

Under standard growth conditions, we observed no significant differences in the growth or morphology between mutant and wild type Col-0 plants. However, mutants seedlings displayed insensitivity to salt as compared to Col-0 (Figures 6C–E). In the presence of 80 mM and 150 mM NaCl, the germination rate of mutant was faster than that of Col-0. Mutants seedlings had enhanced salt stress tolerance and had longer root lengths compared to Col-0 (Figures 6F,G), suggesting that *PPR96* may function in response to salt stress.

### Mutant *ppr96* plants are insensitive to ABA and oxidative stress

**Figure 7:**
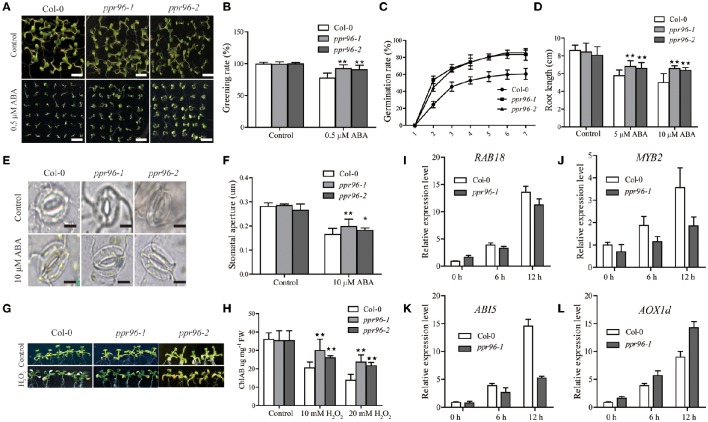
**ABA and oxidative stress insensitivity of ***ppr96*** mutants plants. (A)** Growth of Col-0 and mutants plants on ½ MS medium containing zero or 0.5 μM ABA. Bars = 0.5 cm. **(B)** Comparison of green cotyledon percentages of Col-0 and mutants plants. **(C)** Germination rates of Col-0 and mutants. Seedlings were grown with or without 1 μM ABA. Germination rates were determined daily after stratificaction. Data represent means ± SD (*n* = 108). **(D)** Effects of ABA on root growth of Col-0 and mutants plants. Data represent means ± SD (*n* = 50) from three independent experiments. **(E)** Stomatal movement profiles of Col-0 and mutants plants. Stomatal guard cells were observed in the epidermal peels treated with a solution containing 25 mM KCl and 10 mM MES-Tris (pH 6.15) for 1 h in the light and subsequently treated with 10 μM ABA for 3 h. Bars = 10 μm. **(F)** Stomatal closure of guard cells resulting from ABA treatment. Data are the mean ratios of width to length ± SD of three independent experiments (*n* = 30). **(G)** 2-week old Col-0 and *ppr96* mutants plants were treated with 10 mM H_2_O_2_ for 4 d. **(H)** Reduction of chlorophyll levels in response to H_2_O_2_ treatment during a 4 d period. **(I–L)** Expression levels of *RAB18, MYB2, ABI5*, and *AOX1d* in Col-0 and *ppr96-1* mutant plants under normal conditions and under 1 μM ABA treatment during for 0, 6, and 12 h. Measurement was performed via qPCR. Values represent means ± SD with three biological replicates. Student's *t*-tests were used to generate the *P*-values. ^*^P < 0.05; ^**^P < 0.01.

Assays were performed to examine the ABA insensitivity of *ppr96-1 and ppr96-2*. The germination and root length of *ppr96* mutants seedings were not severely affected by exogenous ABA compared to Col-0 (Figures 7A–D). ABA regulates stomatal closure to avoid water loss during drought stress treatment (Leung and Giraudat, 1998). As shown in Figures 7E,F, treatment of *ppr96-1* and *ppr96-2* leaves with ABA did not cause pronounced stomatal closure, whereas ABA treatment caused obvious stomatal closure in Col-0 leaves. These results showed that the functional deficiency in *ppr96* decreased exogenous ABA sensitivity in the process of stomatal closure, suggesting that *PPR96* is a positive regulator of ABA responses and adjusts exogenous ABA responses in development and stress responses. We also found that *ppr96-1* and *ppr96-2* mutant plants are insensitive to oxidative stress. In the presence of sublethal contents of hydrogen peroxide, the *ppr96-1* and *ppr96-2* mutant displayed relatively slower bleaching and chlorophyll degradation responses as compared to Col-0 plants (Figures 7G,H).

Quantitative real-time PCR (qPCR) was performed to analyze the expression of stress-response marker genes (*RAB18, MYB2*, and *ABI5*) in Col-0 and *ppr96-1* plants under ABA treatment (Figures 7I–L). The results showed that all the stress marker genes had up-regulated expression in Col-0 and *ppr96*-1 under ABA treatment at 6 and 12 h (Figures 7I–L). However, the expression levels of those marker genes in *ppr96*-1 plants were obviously lower than Col-0 plants at 6 and 12 h. Notably, ABA treatment enhanced *AOX1d* transcription in both Col-0 and *ppr96*-1 mutant plants at 0, 6, and 12 h, but the expression level of *AOX1d* gene in *ppr96*-1 plants was higher than Col-0 plants at each time point, and this trend was significantly increased at 12 h as compared to Col-0. AOXs proteins are known to prevent the accumulation of ROS during stress in plants (Navrot et al., 2007). Enhanced induction of *AOX1d* transcription suggested the activation of the compensatory AOX pathway in *ppr96-1* mutant mitochondria. These results suggest that the *PPR96* gene may important function in connecting the regulation of both oxidative respiration and environmental responses in *A. thaliana*.

### PPR96 is localized in the mitochondria

**Figure 8:**
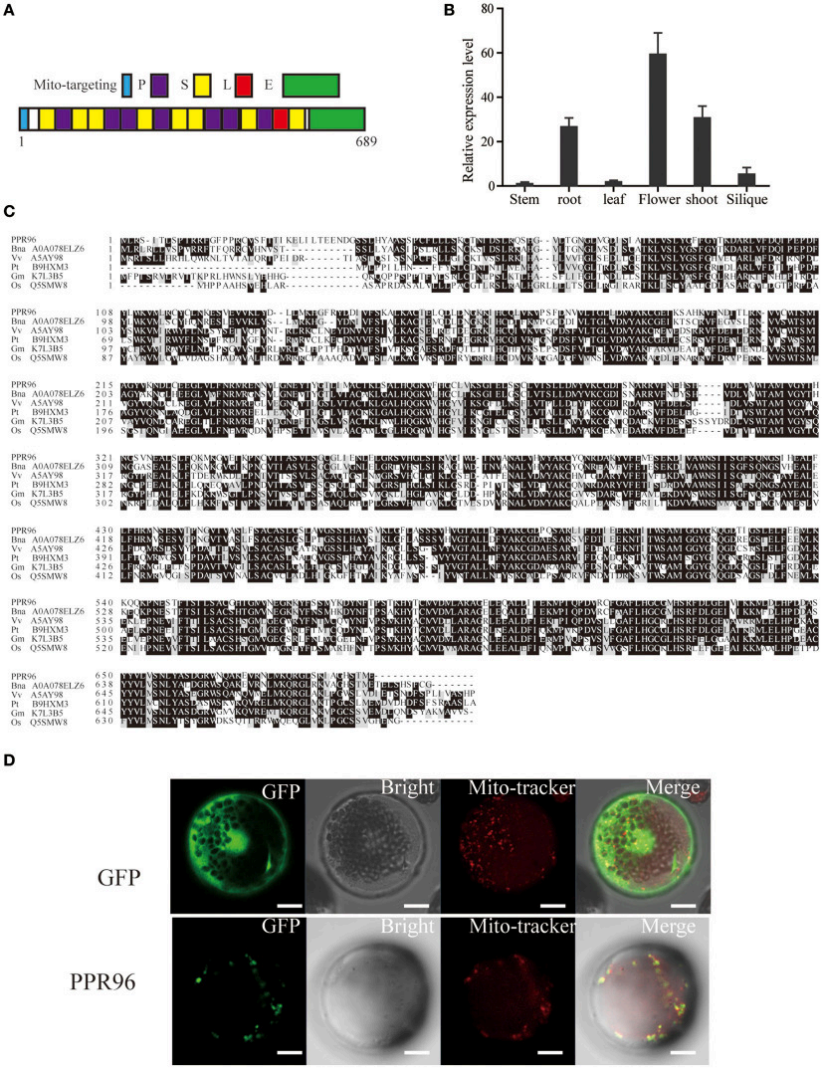
**Main features of the PPR96 protein and its subcellular localization. (A)** Conserved domains of the PPR96 protein as defined by Lurin et al. (2004). **(B)** RT-PCR analysis of the expression pattern of *PPR96*. *ACT2* was used as control. **(C)** Multiple sequence alignment of PPR96 and closely related proteins in other species. Black shading indicates conserved residues, and gray indicates residues identical to PPR96. Proteins were aligned using ClustalW with default gap penalties (Thompson et al., 1997). **(D)** PPR96 is localized to mitochondria. Green fluorescence indicates GFP, red fluorescence indicates stained protoplasts (Mitotracker Orange), and yellow fluorescence indicates images with the two types of fluorescence merged. Bars = 5 μm. Bna, *Brassica napus*; Vv, *Vitis vinifera*; Pt, *Populus trichocarpa*; Os, *Oryza sativa*; Rc, *Ricinus communis*.

The *PPR96* gene has a single exon with an ORF of 2070 bp and putatively encodes a 77-kD PPR96 protein. Analysis of the PPR96 protein sequence by the TargetP program (http://www.cbs.dtu.dk/services/TargetP) (mitochondrial score 0.660), which suggested the PPR96 protein is targeted to mitochondria. The PPR96 protein carries a predicted mitochondrial targeting signal and 16 conserved PPR motifs, and shows the characteristics of the E subgroup of PPR proteins, having a M-63-S-P-S-S-P-P-S-P-S-4-S-P-P-3-S-P-L-S-5-E repeat sequence and a typical domain arrangement (numbers show the number of residues in between domains; Figure 8A). RT-PCR analysis with *PPR96*-specific primers was used to investigate the tissue specific expression pattern of *PPR96*. *PPR96* was detected in roots, flowers, and shoots (Figure 8B). PPR96 orthologs sharing highly conserved domain structures have been identified in *Brassica napus, Vitis vinifera, Populus trichocarpa, Glycine max*, and rice (Figure 8C).

To identify the subcellular localization of PPR96, the full-length *PPR96* was cloned and inserted into a subcellular localization vector that included a GFP protein-encoding gene under the control of the 35S promoter; this construct was transformed into *A. thaliana* protoplasts. To confirm that the putative mitochondrion-targeting PPR96 protein was expressed in mitochondria, we stained transformed *A. thaliana* protoplasts with a mitochondria-specific dye (Jiang et al., 2006) and then observed the samples with 488 and 543 nm illumination. The green fluorescent signals co-localized with the MitoTracker Orange fluorescent signals (Figure 8D). This result revealed that the PPR96-GFP fusion was localized to mitochondria.

## Discussion

RNA editing is a process of RNA maturation involved in the insertion, deletion, or modification of nucleotides (Smith et al., 1997). The RNA editing is more complex in plants than that of other organisms. In organellar transcripts of higher plants, specific cytidine residues are converted into uridine residues. The post-transcriptional conversion of specific cytosines to uracil in mitochondrial and plastid transcripts is unique to land plants (Steinhauser et al., 1999). Thirty-four sites are edited in *Arabidopsis* plastids (Chateigner-Boutin and Small, 2007), whereas more than 450 editing sites are edited in *Arabidopsis* mitochondria (Giegé and Brennicke, 1999; Bentolila et al., 2008; Zehrmann et al., 2008). In many cases, editing results in the restoration of conserved amino acid residues, a process that is essential for protein function in plastids (Bock et al., 1994; Sasaki et al., 2001). The *A. thaliana* genome contains more than 450 PPRs. Most of these are aimed at plastids and/or mitochondria (Lurin et al., 2004), and their functions remain largely sparse (Schmitz-Linneweber and Small, 2008). Of the 450 PPR proteins, the 105 E subgroup members may have a common functions as trans-factors of RNA editing in chloroplasts or mitochondria (Okuda et al., 2007). *A. thaliana* chloroplast editing trans-factors CRR4 and CRR21, for example, can regulate the activity of NAD(P)H dehydrogenase (Kotera et al., 2005; Okuda et al., 2007), and each is required for the editing of a different C target in the *ndhD* transcript. The PPR protein named CLB19 was required for editing of C targets in *rpoA* and *clpP* transcripts (Chateigner-Boutin et al., 2008). *A. thaliana* PPR protein SLO1 is required for RNA editing of *nad4* and *nad9* in mitochondria; its absence affects plant growth and development. In addition, several PPR proteins that contain E/E+ motif are known to be implicated with abiotic stresses in *A. thaliana*. These include: MEF11/LOI1, PGN, AHG1, and SLG1; all of these proteins have been implicated with *A. thaliana* biotic and/or abiotic stress tolerance (Sung et al., 2010; Laluk et al., 2011; Murayama et al., 2012; Yuan and Liu, 2012). The functional link of many PPRs in plastid and mitochondrial development and/or regulation suggests these proteins may play key roles in regulating oxidation balance in cellular redox that are elicited by different types of stress (Lurin et al., 2004; Andrés et al., 2007).

In this paper, the phylogenetic relationships of the E subgroup PPR proteins in eudicot model plant *A. thaliana* and monocots model plant rice were analyzed, revealing that all of the E subgroup PPR proteins can be categorized into five discrete groups (Cluster I to V) (Figure 1). E subgroup PPR proteins in both species showed there generally appears an even mix of genes from both species, excepting Cluster II. The genes in Cluster II may have expanded after the separation of monocots and dicots. Additionally, gene structure and chromosomal distribution analysis showed that 87.6% of the E subgroup PPR genes lacked intron(s) and 14 (13.3%) of the PPR genes were found to be tandem repeats (Figure 2). The *A. thaliana* genome has undergone genome-wide duplication events, including polyploidy, which has great impact on the amplification of members of a gene family in the genome (Seoighe, 2003). Recent research showed that the expansion of PPR gene family prior to the divergence of the euphyllophytes and the lycophytes in land plants, and tandem and segmental duplication are responsible for the expansion of the PPR gene family in vascular plants (Liu et al., 2016). These results suggested that the E subgroup PPR genes may have a common origin in both eudicot model plant *A. thaliana* and monocots model plant rice, and the expansion of this gene family occurred prior to the monocot/dicot divergence in land plants (O'Toole et al., 2008), and the expansion of E subgroup PPRs in *A. thaliana* may results from localized gene duplications.

Microarray analysis of the expression of the E subgroup PPR genes at several stages of development of *A. thaliana* revealed that 25, 23, and 34 of these genes were differentially expressed at least one of the stages of seed imbibition, seed development stage(s), and flowers, respectively (Figure 3, Table S4). Notably, 11, 18, and 17 genes were preferentially expressed in imbibing seeds, SAM, and mature pollen, respectively (Figure 3, Table S4). This suggested that these genes may play important roles in important roles in a wide range of physiological and developmental processes.

Recently, three E subgroup PPR proteins were found to function in the highly complex ABA signaling network. The functions of PGNs in the regulation of ROS homeostasis in mitochondria may occur through the regulation of mitochondria-nucleus retrograde signaling when plants grow under abiotic and biotic stress conditions. (Laluk et al., 2011). SLG1 regulates *nad3* (mitochondrion complex I) transcription by regulating RNA editing events in mitochondria and affecting the expression of genes involved in the alternative respiratory pathway (Yuan and Liu, 2012). In the present study, analysis of publically-available microarray data suggested that several E subgroup PPR genes may participate in responses to abiotic stresses (Figure 4, Table S5). Mutant plants of the mitochondria-localized *A. thaliana PPR96* gene were insensitive to salt, ABA, and oxidative stress. This is the first E subgroup PPR gene mutant identified to be associated with plant resistance to abiotic stresses. We performed a preliminary phenotypic characterization of *ppr96* mutants plants under salt, ABA, and oxidative stress. It is unclear which specific RNA editing sites are deleteriously affected in the *ppr96* mutants. It is not clear whether the loss of *PPR96* function will potentially enhance or decrease ROS accumulation in seedlings in response to abiotic stress. qPCR showed that ABA treatment increased the *AOX1d* transcription level in the *ppr96* mutant as compared to Col-0. *AOX1d* encodes an AOX protein; these proteins are known to capture the excess electrons from ubiquitin to prevent the accumulation of ROS during stress (Navrot et al., 2007). Whether the *ppr96* mutant can induce changes in mitochondrial electron transport also requires further investigation. Enhanced induction of *AOX1d* transcription suggests the activation of the compensatory AOX pathway in *ppr96* mutant mitochondria. These results suggest that PPR96 may play roles in connecting the regulation of both oxidative respiration and environmental adaptation in *A. thaliana*. Additional physiological and biochemical experiments will need to be performed to further explore this supposition.

## Author contributions

ZX coordinated the project, conceived and designed experiments, and edited the manuscript; JL performed experiments, analyzed data, and wrote the first draft of the manuscript; JZ and PL analyzed data; MC provided analytical tools and managed reagents; CG contributed with valuable discussions; YM coordinated the project and contributed with valuable discussions. All authors have read and approved the final version of the manuscript.

### Conflict of interest statement

The authors declare that the research was conducted in the absence of any commercial or financial relationships that could be construed as a potential conflict of interest.

## Supplementary material

Figure S1 **Hierarchial clustering display of 105 E subgroup PPR genes represented on NASCArrays ***A. thaliana*** genome array in various ***A. thaliana*** organs and developmental stages (mentioned at the top of each lane)**. The average log signal values were used for clustering. The color scale (representing log signal values) is shown at the bottom.

Table S1 **The sequences of primers used in the study**. The sequences shown in lower case were added to generate a restriction enzyme site.

Table S2 **E-subgroup PPR genes in ***A. thaliana*****. Detailed genomic information including domain/class present, alias, ORF length, protein length, genomic locus (chromosomal location), number of introns within ORF, subcellular localization, isoelectric point, and molecular weight (kDa) of the PPR proteins for each PPR gene.

Table S3 **Average log signal values of 105 E-subgroup PPR genes from three biological replicates of each sample**.

Table S4 **Average log signal values of 34 differentially expressed E-subgroup PPR genes from three biological replicates of each sample**.

Table S5 **Average log signal values of 105 E-subgroup of PPR genes subjected to salt, cold, heat, drought, osmotic, oxidative, and wounding treatment**. The background color indicates the differentially expressed genes under different stress treatments.
