# Supplementary figures and images for: The E-Subgroup Pentatricopeptide Repeat Protein Family in Arabidopsis thaliana and Confirmation of the Responsiveness PPR96 to Abiotic Stresses
Source: Front Plant Sci. 2016 Dec 5;7:1825. doi: 10.3389/fpls.2016.01825 (PMC5136568; doi:10.3389/fpls.2016.01825)

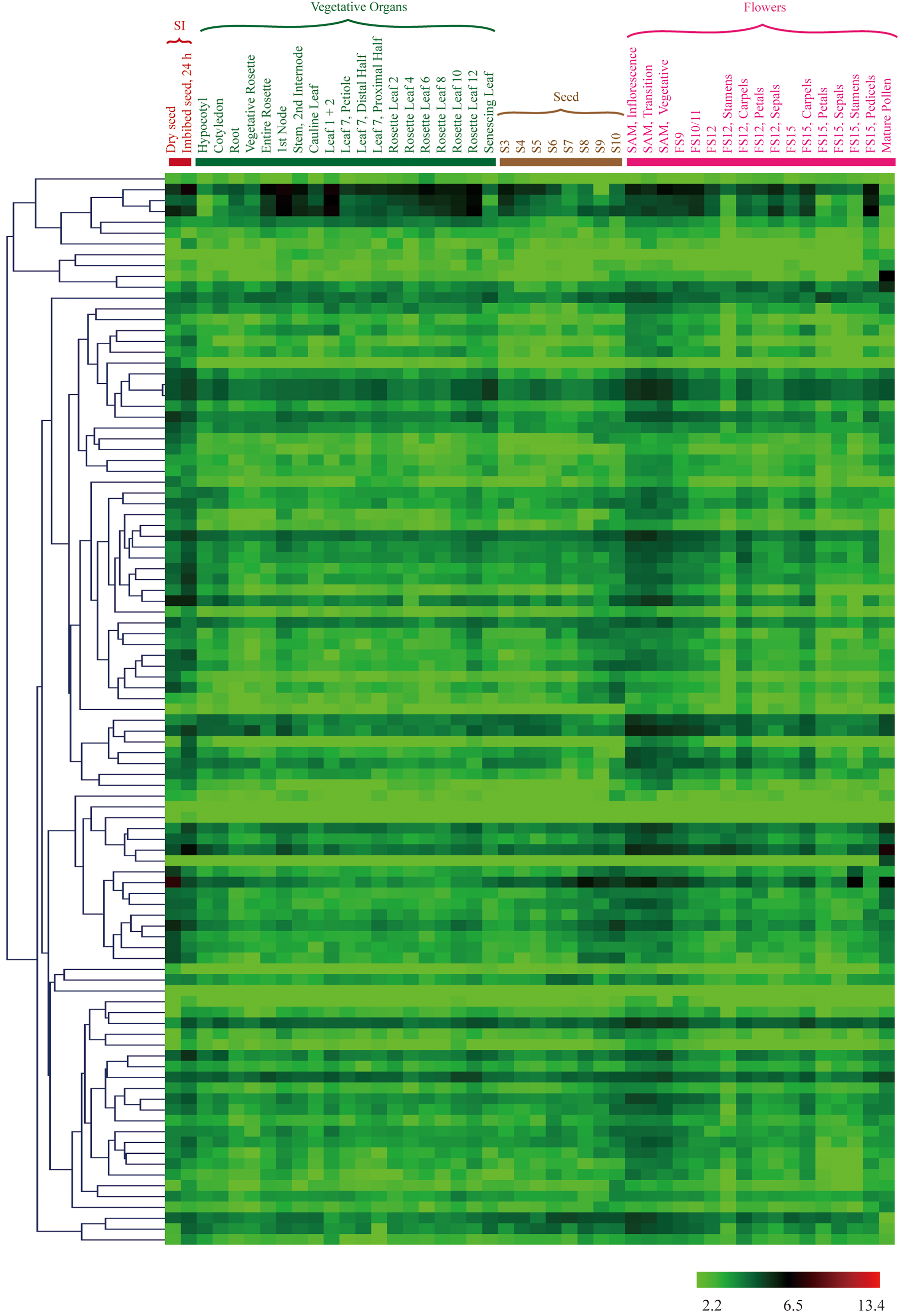

Supplement: Figure S1 — Hierarchial clustering display of 105 E subgroup PPR genes represented on NASCArrays A. thaliana genome array in various A. thaliana organs and developmental stages (mentioned at the top of each lane). The average log signal values were used for clustering. The color scale (representing log signal values) is shown at the bottom. [file Image1.TIF]
