# Supplementary material for: The E-Subgroup Pentatricopeptide Repeat Protein Family in Arabidopsis thaliana and Confirmation of the Responsiveness PPR96 to Abiotic Stresses
Source: Front Plant Sci. 2016 Dec 5;7:1825. doi: 10.3389/fpls.2016.01825 (PMC5136568; doi:10.3389/fpls.2016.01825)
Supplement: Table S1 — The sequences of primers used in the study. The sequences shown in lower case were added to generate a restriction enzyme site. [file Table1.docx]

**Table 1.** The sequences of primers used in the study. The sequences shown in lower case were added to generate a restriction enzyme site.

| **Gene** | **Sequences** | **AGI number** |
| --- | --- | --- |
| **For gene cloning** | | At2G03380 |
| PPR96F | ACAAAACGCTGAGACCAACAAC |  |
| PPR96R | AGCCAAGACAACTAACTTACTGCTATTC |  |
| **For vector construction** | |  |
| PPR96-GFPF | ACTGCAGATGCTACGCTCTATCACTC |  |
| PPR96-GFPR | TGGATCCTTCCATTGTGCTATGGCCTGCA |  |
| **For quantitative RT-PCR** | |  |
| RT-PPR96F | GGCAATCTCACGGAGTTTTAA |  |
| RT-PPR96R | TGAAGCTCAGTACACGCTTTCA |  |
| RT-At1g28690F | TGCTTGTTCACATTCTGGTTTA |  |
| RT-At1g28690R | TCACATTGTCCCATTTATCATTAG |  |
| RT-At3g05340F | CACCTTCCTTCTTGCTTATCTACC |  |
| RT-At3g05340R | GGCATCAACTAACTTCCCACAT |  |
| RT-At3g22150F | CCCGTCTTAGCAAAATCTGTC |  |
| RT-At3g22150R | CCATTCTCATCATTATCCCAAA |  |
| RT-At3g29230F | GCTCAGATAATCCGACGAAAC |  |
| RT-At3g29230R | CTAACACCCAAACCACCACA |  |
| RT-At5g55740F | TCTTCCTTTCAACACTATCCCG |  |
| RT-At5g55740R | AGCATCTCAACAAAACCCATTA |  |
| RT-At5g56310F | AATGCGTTGTTAGCAGGGTA |  |
| RT-At5g56310R | CTGCGATTATAGTCGTCCAAGT |  |
| RT-At5g59200F | CAAGATGCTTTCGTTGTTTTCG |  |
| RT-At5g59200R | TTCCCCAGATTTCCCGTAGA |  |
| ACT2F | GGTAACATTGTGCTCAGTGGTGG | At3g18780 |
| ACT2R | AACGACCTTAATCTTCATGCTGC |  |
| RAB18F | CAGCAGCAGTATGACGAGTA | At5g66400 |
| RAB18R | CAGTTCCAAAGCCTTCAGTC |  |
| RD29AF | ATCACTTGGCTCCACTGTTGTTC | At5g52310 |
| RD29AR | ACAAAACACACATAAACATCCAAAGT |  |
| RD22F | GCGATTGCGGCTGATTTAAC | At5g25610 |
| RD22R | TGGGAATGGGAGTGTTTGGT |  |
| MYB2F | GGCAATAGGTGGTCGAAGATTGCG | At2g47190 |
| MYB2R | GTGTTTGGCTTGCTTTTGGACTCG |  |
| ABI5F | ATTGGCGGAGTTGGAGAGGAAGAG | At2g36270 |
| ABI5R | TCGGTTGTGCCCTTGACTTCAAA |  |
| AOX1D | TACCGCACTCTTCGAC | At1g32350 |
| AOX1D | GGCTGGTTATTCCCACT |  |
| **For verify T-DNA insertion mutants** | | |
| *ppr96-1*_LP1 | AGTCCCTGGTTGTGTGCTATG |  |
| *ppr96-1*_RP1 | TCTGGTTCAGGAATTTGATCG |  |
| *ppr96-2*_LP2 | CTGCAATCTTGCTCAGACCTC |  |
| *ppr96-2*_RP2 | TCGGGTGTTTAATGAGCATTC |  |
| LBb1.3 | ATTTTGCCGATTTCGGAAC |  |
